# 4-[3,5-Bis(ethoxy­carbon­yl)-2,6-dimethyl-4-pyrid­yl]pyridinium nitrate

**DOI:** 10.1107/S1600536810015035

**Published:** 2010-04-30

**Authors:** Yumei Li

**Affiliations:** a Department of Chemistry, Dezhou University, Shandong 253023, People’s Republic of China

## Abstract

In the title mol­ecular salt, C_18_H_21_N_2_O_4_^+^·NO_3_^−^, the dihedral angle between the two pyridine rings is 61.24 (8)°. In the crystal, the cation and anion are linked by inter­molecular N—H⋯O hydrogen bonds.

## Related literature

For general background to metal-organic frameworks, see: Zhang *et al.* (2003 ▶).
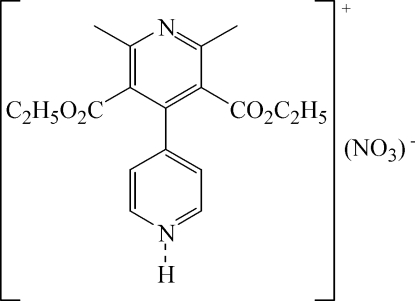


## Experimental

### 

#### Crystal data

C_18_H_21_N_2_O_4_^+^·NO_3_^−^
*M* *_r_* = 391.38
Orthorhombic, 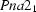

*a* = 9.075 (9) Å
*b* = 15.496 (15) Å
*c* = 14.125 (13) Å
*V* = 1987 (3) Å^3^
*Z* = 4
Mo *K*α radiation
μ = 0.10 mm^−1^
*T* = 296 K
0.37 × 0.33 × 0.24 mm

#### Data collection

Bruker APEXII CCD diffractometer
Absorption correction: multi-scan (*SADABS*; Bruker, 2001 ▶) *T*_min_ = 0.963, *T*_max_ = 0.975
9196 measured reflections
3395 independent reflections
2877 reflections with *I* > 2σ(*I*)
*R*_int_ = 0.156

#### Refinement

*R*[*F* ^2^ > 2σ(*F* ^2^)] = 0.043
*wR*(*F* ^2^) = 0.115
*S* = 1.00
3395 reflections
258 parameters
1 restraint
H-atom parameters constrained
Δρ_max_ = 0.17 e Å^−3^
Δρ_min_ = −0.21 e Å^−3^

### 

Data collection: *APEX2* (Bruker, 2004 ▶); cell refinement: *SAINT-Plus* (Bruker, 2001 ▶); data reduction: *SAINT-Plus*; program(s) used to solve structure: *SHELXS97* (Sheldrick, 2008 ▶); program(s) used to refine structure: *SHELXL97* (Sheldrick, 2008 ▶); molecular graphics: *SHELXTL* (Sheldrick, 2008 ▶); software used to prepare material for publication: *SHELXTL*.

## Supplementary Material

Crystal structure: contains datablocks global, I. DOI: 10.1107/S1600536810015035/hb5413sup1.cif

Structure factors: contains datablocks I. DOI: 10.1107/S1600536810015035/hb5413Isup2.hkl

Additional supplementary materials:  crystallographic information; 3D view; checkCIF report

## Figures and Tables

**Table 1:** Hydrogen-bond geometry (Å, °)

*D*—H⋯*A*	*D*—H	H⋯*A*	*D*⋯*A*	*D*—H⋯*A*
N2—H2*A*⋯O5^i^	0.86	1.90	2.752 (3)	171

Symmetry code: (i) 
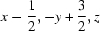
.

**Fig. 1.**
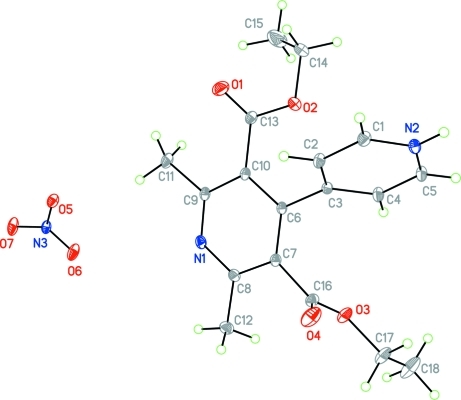
The moleular structure of (I) with displacement ellipsoids drawn at the 30% probability level; H atoms are given as spheres of arbitrary radius.

## supplementary crystallographic information

### Comment

In recent years, the design and construction of metal-organic frameworks through the coordination of metal ions with multifunctional organic ligands have received extensive attention due to their impressive structural diversities in architectures and their potential applications as functional materials (Zhang *et al.*, 2003). Whereas, it is more important to design the novel organic ligand. Here, we describe the recystallization and structural characterization of the title compound.

The molecular structure is shown in Fig 1. The dihedral angle between the two pyridine rings is 61.24 (8) °. N—H···O and N—H···N hydrogen bonding between the cations and anions leads to a consolidation of the structure (Fig. 2; Table 1).

### Experimental

A mixture of 2,6-dimethyl-4-(4-pyridyl)pyridine-3,5-dicarboxylate (1 mmol, 0.39 g) and ammonium nitrate (2 mmol, 0.16 g) in 20 ml ethanol was refluxed for half an hour. The obtained filtrate was evaporated in one open flask at room temperature. One week later, yellow blocks of (I) were obained. Anal. C_20_H_22_NO_7_: C, 55.61; H, 5.41; N, 7.21 %. Found: C, 55.56; H, 5.33; N, 7.10 %.

### Refinement

The absolute structure of (I) is indeterminate based on the present model. All hydrogen atoms bound to aromatic carbon atoms were refined in calculated positions using a riding model with a C—H distance of 0.93 Å and *U*_iso_ = 1.2*U*_eq_(C). Hydrogen atoms attached to aromatic N atoms were refined with a N—H distance of 0.86 Å and *U*_iso_ = 1.2*U*_eq_(N).

### Crystal data

**Table:**

C_18_H_21_N_2_O_4_^+^·NO_3_^−^	*F*(000) = 824
*M**_r_* = 391.38	*D*_x_ = 1.309 Mg m^−^^3^
Orthorhombic, *P**n**a*2_1_	Mo *K*α radiation, λ = 0.71073 Å
Hall symbol: P 2c -2n	Cell parameters from 3948 reflections
*a* = 9.075 (9) Å	θ = 2.2–25.9°
*b* = 15.496 (15) Å	µ = 0.10 mm^−^^1^
*c* = 14.125 (13) Å	*T* = 296 K
*V* = 1987 (3) Å^3^	Block, yellow
*Z* = 4	0.37 × 0.33 × 0.24 mm

### Data collection

**Table:**

Bruker APEXII CCD diffractometer	3395 independent reflections
Radiation source: fine-focus sealed tube	2877 reflections with *I* > 2σ(*I*)
graphite	*R*_int_ = 0.156
phi and ω scans	θ_max_ = 25.0°, θ_min_ = 2.0°
Absorption correction: multi-scan (*SADABS*; Bruker, 2001)	*h* = −10→10
*T*_min_ = 0.963, *T*_max_ = 0.975	*k* = −18→10
9196 measured reflections	*l* = −15→16

### Refinement

**Table:**

Refinement on *F*^2^	Secondary atom site location: difference Fourier map
Least-squares matrix: full	Hydrogen site location: inferred from neighbouring sites
*R*[*F*^2^ > 2σ(*F*^2^)] = 0.043	H-atom parameters constrained
*wR*(*F*^2^) = 0.115	*w* = 1/[σ^2^(*F*_o_^2^) + (0.069*P*)^2^] where *P* = (*F*_o_^2^ + 2*F*_c_^2^)/3
*S* = 1.00	(Δ/σ)_max_ = 0.001
3395 reflections	Δρ_max_ = 0.17 e Å^−^^3^
258 parameters	Δρ_min_ = −0.20 e Å^−^^3^
1 restraint	Extinction correction: *SHELXL97* (Sheldrick, 2008), Fc^*^=kFc[1+0.001xFc^2^λ^3^/sin(2θ)]^-1/4^
Primary atom site location: structure-invariant direct methods	Extinction coefficient: 0.0134 (12)

### Special details

**Table:**

Geometry. All esds (except the esd in the dihedral angle between two l.s. planes) are estimated using the full covariance matrix. The cell esds are taken into account individually in the estimation of esds in distances, angles and torsion angles; correlations between esds in cell parameters are only used when they are defined by crystal symmetry. An approximate (isotropic) treatment of cell esds is used for estimating esds involving l.s. planes.
Refinement. Refinement of *F*^2^ against ALL reflections. The weighted *R*-factor wR and goodness of fit *S* are based on *F*^2^, conventional *R*-factors *R* are based on *F*, with *F* set to zero for negative *F*^2^. The threshold expression of *F*^2^ > σ(*F*^2^) is used only for calculating *R*-factors(gt) etc. and is not relevant to the choice of reflections for refinement. *R*-factors based on *F*^2^ are statistically about twice as large as those based on *F*, and *R*- factors based on ALL data will be even larger.

### Fractional atomic coordinates and isotropic or equivalent isotropic displacement parameters (Å^2^)

**Table:**

	*x*	*y*	*z*	*U*_iso_*/*U*_eq_	
C1	−0.10369 (19)	1.05766 (11)	0.20140 (17)	0.0498 (5)	
H1	−0.2022	1.0738	0.1979	0.060*	
C2	−0.06618 (18)	0.97534 (10)	0.22947 (15)	0.0460 (5)	
H2	−0.1393	0.9361	0.2460	0.055*	
C3	0.08043 (17)	0.95128 (10)	0.23295 (14)	0.0372 (4)	
C4	0.18687 (19)	1.01225 (10)	0.20996 (15)	0.0476 (5)	
H4	0.2863	0.9980	0.2128	0.057*	
C5	0.14389 (18)	1.09410 (11)	0.18284 (16)	0.0496 (5)	
H5	0.2145	1.1352	0.1671	0.060*	
C6	0.12219 (17)	0.86086 (9)	0.26006 (13)	0.0352 (4)	
C7	0.20576 (18)	0.84398 (10)	0.34073 (13)	0.0389 (4)	
C8	0.24140 (19)	0.75802 (10)	0.36386 (14)	0.0420 (4)	
C9	0.10982 (18)	0.70663 (10)	0.23457 (14)	0.0404 (4)	
C10	0.07328 (16)	0.79095 (9)	0.20499 (13)	0.0365 (4)	
C11	0.0619 (2)	0.62888 (10)	0.1782 (2)	0.0585 (6)	
H11A	0.0733	0.5778	0.2161	0.088*	
H11B	0.1214	0.6241	0.1223	0.088*	
H11C	−0.0397	0.6352	0.1605	0.088*	
C12	0.3328 (3)	0.73555 (13)	0.44732 (18)	0.0596 (6)	
H12A	0.3463	0.6742	0.4498	0.089*	
H12B	0.2842	0.7546	0.5039	0.089*	
H12C	0.4270	0.7633	0.4422	0.089*	
C13	−0.00568 (19)	0.80402 (10)	0.11383 (15)	0.0428 (5)	
C14	0.0243 (3)	0.85562 (17)	−0.0438 (2)	0.0784 (7)	
H14A	0.0605	0.9081	−0.0731	0.094*	
H14B	−0.0825	0.8571	−0.0447	0.094*	
C15	0.0777 (6)	0.77990 (19)	−0.0969 (3)	0.1235 (14)	
H15A	0.1834	0.7783	−0.0949	0.185*	
H15B	0.0455	0.7838	−0.1615	0.185*	
H15C	0.0388	0.7282	−0.0689	0.185*	
C16	0.2496 (2)	0.91621 (11)	0.40602 (15)	0.0482 (5)	
C17	0.4449 (3)	1.00763 (16)	0.4561 (2)	0.0846 (8)	
H17A	0.3935	1.0611	0.4428	0.102*	
H17B	0.4291	0.9931	0.5221	0.102*	
C18	0.5981 (3)	1.0179 (2)	0.4383 (3)	0.1241 (13)	
H18A	0.6478	0.9642	0.4494	0.186*	
H18B	0.6373	1.0613	0.4798	0.186*	
H18C	0.6126	1.0352	0.3737	0.186*	
N3	0.53347 (15)	0.16998 (8)	0.17481 (14)	0.0488 (4)	
N1	0.19326 (16)	0.69168 (8)	0.31060 (12)	0.0440 (4)	
N2	0.00187 (16)	1.11408 (8)	0.17919 (14)	0.0484 (4)	
H2A	−0.0231	1.1652	0.1619	0.058*	
O1	−0.12303 (17)	0.77548 (12)	0.09504 (13)	0.0770 (5)	
O2	0.07701 (15)	0.85022 (8)	0.05417 (12)	0.0595 (4)	
O3	0.38730 (15)	0.93821 (9)	0.39511 (13)	0.0643 (4)	
O4	0.16624 (19)	0.94931 (12)	0.46120 (15)	0.0888 (6)	
O5	0.43298 (14)	0.21643 (8)	0.14155 (13)	0.0615 (4)	
O6	0.64708 (15)	0.20368 (9)	0.20110 (18)	0.0856 (6)	
O7	0.51772 (17)	0.09067 (8)	0.17934 (19)	0.0873 (6)	

### Atomic displacement parameters (Å^2^)

**Table:**

	*U*^11^	*U*^22^	*U*^33^	*U*^12^	*U*^13^	*U*^23^
C1	0.0446 (8)	0.0371 (9)	0.0676 (15)	0.0042 (7)	−0.0064 (9)	−0.0048 (9)
C2	0.0444 (8)	0.0322 (8)	0.0613 (13)	−0.0021 (7)	0.0003 (9)	−0.0025 (9)
C3	0.0448 (8)	0.0281 (7)	0.0388 (10)	−0.0001 (6)	−0.0026 (7)	−0.0020 (7)
C4	0.0435 (8)	0.0332 (8)	0.0660 (14)	−0.0010 (6)	0.0001 (9)	0.0057 (8)
C5	0.0494 (8)	0.0338 (8)	0.0657 (14)	−0.0061 (7)	0.0023 (10)	0.0049 (9)
C6	0.0384 (7)	0.0260 (7)	0.0411 (11)	0.0003 (6)	0.0040 (7)	−0.0009 (7)
C7	0.0420 (8)	0.0316 (7)	0.0429 (11)	0.0000 (6)	0.0031 (8)	−0.0030 (7)
C8	0.0477 (8)	0.0370 (8)	0.0414 (11)	0.0053 (7)	0.0047 (8)	0.0016 (8)
C9	0.0439 (8)	0.0279 (7)	0.0495 (12)	−0.0014 (6)	0.0080 (8)	−0.0044 (7)
C10	0.0386 (7)	0.0275 (7)	0.0434 (11)	−0.0017 (6)	0.0034 (7)	−0.0042 (7)
C11	0.0677 (11)	0.0310 (8)	0.0768 (16)	−0.0052 (7)	−0.0037 (12)	−0.0144 (9)
C12	0.0761 (13)	0.0506 (11)	0.0519 (14)	0.0082 (9)	−0.0100 (11)	0.0058 (10)
C13	0.0418 (8)	0.0355 (8)	0.0513 (12)	0.0007 (7)	−0.0003 (8)	−0.0081 (8)
C14	0.0979 (16)	0.0791 (14)	0.0582 (17)	−0.0109 (13)	−0.0182 (13)	0.0275 (13)
C15	0.211 (4)	0.095 (2)	0.064 (2)	0.013 (2)	−0.011 (3)	0.0004 (19)
C16	0.0559 (10)	0.0384 (8)	0.0502 (12)	0.0046 (7)	−0.0052 (9)	−0.0088 (9)
C17	0.0961 (17)	0.0685 (13)	0.089 (2)	−0.0206 (13)	−0.0115 (15)	−0.0356 (13)
C18	0.0940 (18)	0.126 (2)	0.153 (3)	−0.0447 (18)	0.001 (2)	−0.069 (2)
N3	0.0451 (7)	0.0320 (6)	0.0694 (12)	−0.0002 (6)	0.0099 (8)	−0.0050 (8)
N1	0.0547 (8)	0.0281 (6)	0.0493 (10)	0.0016 (6)	0.0050 (7)	0.0034 (7)
N2	0.0615 (9)	0.0258 (6)	0.0578 (11)	0.0058 (6)	−0.0071 (8)	0.0018 (7)
O1	0.0578 (8)	0.1056 (12)	0.0677 (12)	−0.0226 (8)	−0.0104 (8)	−0.0015 (9)
O2	0.0662 (8)	0.0590 (7)	0.0533 (10)	−0.0079 (7)	−0.0060 (7)	0.0137 (7)
O3	0.0663 (8)	0.0602 (7)	0.0664 (11)	−0.0137 (6)	−0.0004 (8)	−0.0270 (8)
O4	0.0803 (10)	0.0871 (10)	0.0990 (14)	0.0011 (8)	0.0135 (10)	−0.0543 (10)
O5	0.0521 (7)	0.0398 (6)	0.0925 (13)	0.0035 (5)	−0.0088 (7)	−0.0036 (7)
O6	0.0535 (7)	0.0432 (7)	0.1601 (19)	−0.0011 (6)	−0.0234 (10)	0.0018 (10)
O7	0.0748 (8)	0.0284 (6)	0.159 (2)	−0.0030 (6)	0.0012 (12)	0.0025 (9)

### Geometric parameters (Å, °)

**Table:**

C1—N2	1.334 (2)	C12—H12B	0.9600
C1—C2	1.379 (3)	C12—H12C	0.9600
C1—H1	0.9300	C13—O1	1.183 (2)
C2—C3	1.383 (3)	C13—O2	1.336 (2)
C2—H2	0.9300	C14—O2	1.466 (3)
C3—C4	1.390 (2)	C14—C15	1.474 (5)
C3—C6	1.501 (2)	C14—H14A	0.9700
C4—C5	1.381 (3)	C14—H14B	0.9700
C4—H4	0.9300	C15—H15A	0.9600
C5—N2	1.327 (3)	C15—H15B	0.9600
C5—H5	0.9300	C15—H15C	0.9600
C6—C7	1.394 (3)	C16—O4	1.201 (3)
C6—C10	1.406 (2)	C16—O3	1.304 (3)
C7—C8	1.409 (3)	C17—C18	1.422 (4)
C7—C16	1.504 (3)	C17—O3	1.474 (3)
C8—N1	1.347 (2)	C17—H17A	0.9700
C8—C12	1.483 (3)	C17—H17B	0.9700
C9—N1	1.334 (3)	C18—H18A	0.9600
C9—C10	1.411 (2)	C18—H18B	0.9600
C9—C11	1.508 (3)	C18—H18C	0.9600
C10—C13	1.488 (3)	N3—O6	1.214 (2)
C11—H11A	0.9600	N3—O7	1.239 (2)
C11—H11B	0.9600	N3—O5	1.253 (2)
C11—H11C	0.9600	N2—H2A	0.8600
C12—H12A	0.9600		
			
N2—C1—C2	119.78 (16)	H12B—C12—H12C	109.5
N2—C1—H1	120.1	O1—C13—O2	124.4 (2)
C2—C1—H1	120.1	O1—C13—C10	125.22 (19)
C1—C2—C3	119.82 (16)	O2—C13—C10	110.38 (15)
C1—C2—H2	120.1	O2—C14—C15	109.1 (2)
C3—C2—H2	120.1	O2—C14—H14A	109.9
C2—C3—C4	118.51 (16)	C15—C14—H14A	109.9
C2—C3—C6	120.24 (14)	O2—C14—H14B	109.9
C4—C3—C6	121.24 (15)	C15—C14—H14B	109.9
C5—C4—C3	119.53 (16)	H14A—C14—H14B	108.3
C5—C4—H4	120.2	C14—C15—H15A	109.5
C3—C4—H4	120.2	C14—C15—H15B	109.5
N2—C5—C4	119.97 (15)	H15A—C15—H15B	109.5
N2—C5—H5	120.0	C14—C15—H15C	109.5
C4—C5—H5	120.0	H15A—C15—H15C	109.5
C7—C6—C10	118.67 (14)	H15B—C15—H15C	109.5
C7—C6—C3	121.41 (14)	O4—C16—O3	124.64 (19)
C10—C6—C3	119.90 (16)	O4—C16—C7	123.29 (18)
C6—C7—C8	119.46 (15)	O3—C16—C7	112.07 (16)
C6—C7—C16	120.38 (15)	C18—C17—O3	109.0 (2)
C8—C7—C16	120.04 (17)	C18—C17—H17A	109.9
N1—C8—C7	121.18 (18)	O3—C17—H17A	109.9
N1—C8—C12	116.50 (16)	C18—C17—H17B	109.9
C7—C8—C12	122.32 (17)	O3—C17—H17B	109.9
N1—C9—C10	122.17 (15)	H17A—C17—H17B	108.3
N1—C9—C11	116.72 (16)	C17—C18—H18A	109.5
C10—C9—C11	121.04 (18)	C17—C18—H18B	109.5
C6—C10—C9	118.39 (17)	H18A—C18—H18B	109.5
C6—C10—C13	121.75 (15)	C17—C18—H18C	109.5
C9—C10—C13	119.71 (15)	H18A—C18—H18C	109.5
C9—C11—H11A	109.5	H18B—C18—H18C	109.5
C9—C11—H11B	109.5	O6—N3—O7	120.59 (16)
H11A—C11—H11B	109.5	O6—N3—O5	119.06 (15)
C9—C11—H11C	109.5	O7—N3—O5	120.34 (16)
H11A—C11—H11C	109.5	C9—N1—C8	120.07 (14)
H11B—C11—H11C	109.5	C5—N2—C1	122.38 (15)
C8—C12—H12A	109.5	C5—N2—H2A	118.8
C8—C12—H12B	109.5	C1—N2—H2A	118.8
H12A—C12—H12B	109.5	C13—O2—C14	116.27 (18)
C8—C12—H12C	109.5	C16—O3—C17	117.49 (18)
H12A—C12—H12C	109.5		
			
N2—C1—C2—C3	−1.1 (3)	C11—C9—C10—C6	−179.48 (17)
C1—C2—C3—C4	1.6 (3)	N1—C9—C10—C13	173.11 (15)
C1—C2—C3—C6	−177.80 (19)	C11—C9—C10—C13	−3.8 (2)
C2—C3—C4—C5	−1.1 (3)	C6—C10—C13—O1	−124.4 (2)
C6—C3—C4—C5	178.22 (19)	C9—C10—C13—O1	60.1 (3)
C3—C4—C5—N2	0.2 (3)	C6—C10—C13—O2	57.8 (2)
C2—C3—C6—C7	−117.5 (2)	C9—C10—C13—O2	−117.67 (17)
C4—C3—C6—C7	63.1 (3)	C6—C7—C16—O4	76.4 (3)
C2—C3—C6—C10	60.7 (3)	C8—C7—C16—O4	−99.8 (2)
C4—C3—C6—C10	−118.7 (2)	C6—C7—C16—O3	−103.4 (2)
C10—C6—C7—C8	1.3 (2)	C8—C7—C16—O3	80.4 (2)
C3—C6—C7—C8	179.50 (16)	C10—C9—N1—C8	2.3 (3)
C10—C6—C7—C16	−174.90 (16)	C11—C9—N1—C8	179.39 (17)
C3—C6—C7—C16	3.3 (2)	C7—C8—N1—C9	−0.2 (3)
C6—C7—C8—N1	−1.6 (3)	C12—C8—N1—C9	179.67 (17)
C16—C7—C8—N1	174.62 (16)	C4—C5—N2—C1	0.3 (3)
C6—C7—C8—C12	178.55 (17)	C2—C1—N2—C5	0.1 (3)
C16—C7—C8—C12	−5.3 (3)	O1—C13—O2—C14	−7.9 (3)
C7—C6—C10—C9	0.7 (2)	C10—C13—O2—C14	169.90 (17)
C3—C6—C10—C9	−177.59 (15)	C15—C14—O2—C13	−85.5 (3)
C7—C6—C10—C13	−174.87 (15)	O4—C16—O3—C17	1.1 (3)
C3—C6—C10—C13	6.9 (2)	C7—C16—O3—C17	−179.12 (19)
N1—C9—C10—C6	−2.5 (2)	C18—C17—O3—C16	176.0 (3)

### Hydrogen-bond geometry (Å, °)

**Table:**

*D*—H···*A*	*D*—H	H···*A*	*D*···*A*	*D*—H···*A*
N2—H2A···O5^i^	0.86	1.90	2.752 (3)	171

Symmetry codes: (i) *x*−1/2, −*y*+3/2, *z*.
